# Can endurance exercise preconditioning prevention disuse muscle atrophy?

**DOI:** 10.3389/fphys.2015.00063

**Published:** 2015-03-11

**Authors:** Michael P. Wiggs

**Affiliations:** Department of Applied Physiology and Kinesiology, Center for Exercise Science, University of FloridaGainesville, FL, USA

**Keywords:** bed rest, unloading, immobilization, superoxide dismutase, HSP70, PGC1-α, mitophagy, oxidative stress

## Abstract

Emerging evidence suggests that exercise training can provide a level of protection against disuse muscle atrophy. Endurance exercise training imposes oxidative, metabolic, and heat stress on skeletal muscle which activates a variety of cellular signaling pathways that ultimately leads to the increased expression of proteins that have been demonstrated to protect muscle from inactivity –induced atrophy. This review will highlight the effect of exercise-induced oxidative stress on endogenous enzymatic antioxidant capacity (i.e., superoxide dismutase, glutathione peroxidase, and catalase), the role of oxidative and metabolic stress on PGC1-α, and finally highlight the effect heat stress and HSP70 induction. Finally, this review will discuss the supporting scientific evidence that these proteins can attenuate muscle atrophy through exercise preconditioning.

## Introduction

Prolonged periods of inactivity results in a loss of muscle protein, fiber atrophy, and impaired muscle function. It is widely recognized that maintaining skeletal muscle structure and function is important in promoting health and quality of life (Wolfe, 2006). A loss of muscle mass can impair the ability to perform activities of daily living, prolong periods of rehabilitation and be a major risk factor for chronic disease. Therefore, it is important to find ways to attenuate rapid muscle atrophy during periods of prolonged disuse.

Common disuse atrophy conditions include prolonged bed rest, limb immobilization/casting, and prolonged exposure to microgravity. The rapid decrease in muscle size observed during disuse is due to both a reduction in muscle protein synthesis and an increase in protein degradation. Therefore, countermeasures to prevent muscle wasting generally target these pathways. In this regard, it is well established that exercise training is able to increase muscle protein synthesis and has been used as a counter-measure to disuse muscle atrophy in many of these conditions (reviewed in Glover and Phillips, 2010). Indeed, both resistance and endurance exercise have been shown to attenuate muscle disuse muscle atrophy in both human and animal models of muscle atrophy and this has been termed “exercise preconditioning.”

However, not all disuse atrophy conditions allow for exercise training during the period of muscle inactivity. For example, during casting and limb immobilization, it is physically impossible to exercise the affected muscles. Another example is mechanical ventilation induced-diaphragm atrophy. This is caused by inactivity of the diaphragm during mechanical ventilation (MV), which causes rapid (e.g., within 12 h) atrophy of the diaphragm (Powers et al., 2013). Recently our lab demonstrated that endurance exercise training prior to MV protected the diaphragm from muscle atrophy (Smuder et al., 2012a). To date, this is one of the few studies to examine the protective effects of exercise preconditioning against disuse muscle atrophy. Therefore, this review will highlight several potential cytoprotective proteins that are altered with exercise training that may be critical in protecting against disuse muscle atrophy. The review will begin with a brief discussion the regulation of muscle size, followed by the pertinent adaptations related to mitochondria, since mitochondrial health plays a key role in maintaining muscle size and function, and finaly the potential cytoprotective proteins induced by exercise will be discussed.

## Overview of the regulation of muscle size

Muscle atrophy results from decrease in protein synthesis and an increase in protein degradation that ultimately leads to a decrease in myofiber size due to loss of contractile proteins, organelles, nuclei, and cytoplasm (Thomason and Booth, 1990). Indeed, animal studies have demonstrated that an increase in protein degradation and a decrease in protein synthesis contribute to disuse atrophy. This section will briefly describe the molecular basis for these processes (detailed review by Schiaffino et al., 2013).

### Skeletal muscle proteolysis

**Figure 1:**
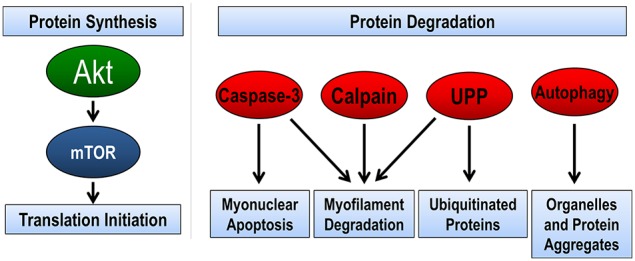
**Pathways regulating skeletal muscle size**. The balance between protein synthesis and protein degradation determines muscle size. Muscle protein synthesis is regulated through the Akt/mTOR pathway. There are four pathways of protein degradation to degrade cellular components, caspase-3 removes nuclei (apoptosis) and along with the calpain family of proteins can breakdown the contractile proteins (myofilaments) in muscle into smaller polypeptides. The ubiquitin-proteasome pathway (UPP) breaks down the small peptides and any other protein targeted by ubiquitination for degradation. Finally, autophagy breaks down organelles, such as mitochondria and endoplasmic reticulum, and large protein aggregates.

In regards to proteolysis, skeletal muscle utilizes four complementary pathways to remove damaged, misfolded, or unnecessary proteins (Figure 1). These pathways include calpain, caspase-3, ubiquitin proteasome pathway, and autophagy (reviewed in Jackman and Kandarian, 2004). The calpain family of proteins is calcium dependent proteases that are important in the initiation of the breakdown of actin, myosin, and other structural proteins. Indeed, target pharmacological inhibition of calpain protects against disuse muscle atrophy (Tischler et al., 1990; Goll et al., 2003; Maes et al., 2007; Nelson et al., 2012; Talbert et al., 2013a). Caspase-3 is a member of the cysteine-aspartic acid protease family. Caspase-3 is most often defined by its central role in the removal of nuclei by myonuclear apoptosis; however, recent info also demonstrates the caspase-3 can work in concert with calpain in the cleavage of myofibrillar proteins (Du et al., 2004; Dupont-Versteegden, 2006; Smuder et al., 2010). Similar to calpain, inhibition or genetic knockdown of caspase-3 is sufficient to attenuate disuse muscle atrophy (McClung et al., 2007; Nelson et al., 2012; Talbert et al., 2013a; Zhu et al., 2013). Together, it is believed that calpain and caspase-3 are required for disuse atrophy because they begin the initial breakdown of the contractile apparatus of the muscle (Jackman and Kandarian, 2004). Further breakdown of these proteins is accomplished by the ubiquitin-proteasome pathway (Lecker et al., 1999). The proteasome pathway is also important in the degradation of misfolded, smaller polypeptides, and unnecessary proteins. Presently, the role of autophagy in disuse atrophy remains unclear. Early studies suggested that pharmacological inhibition of autophagy had minimal effects on muscle atrophy (Tischler et al., 1990); however, recent evidence suggests that autophagy may play an important role in disuse atrophy, specifically in the ability to selectively degrade organelles such as mitochondria (i.e., mitophagy) (Sandri, 2013). This will be discussed in greater detail in later sections.

### Skeletal muscle protein synthesis

Protein synthesis in cells is accomplished by a tightly controlled and structured scheme of signaling pathways, which culminate in the translation of messenger RNA (mRNA) into a specific protein. Within hours of disuse, muscle protein synthesis is rapidly depressed roughly 25–50% of control and reaches a new baseline level that persists throughout the period of inactivity (De Boer et al., 2007; Kortebein et al., 2008; Symons et al., 2009; Ferrando et al., 2010) The Akt/mTOR pathway is a crucial regulator of muscle mass by controlling formation of translation initiation machinery, the rate limiting step in protein synthesis (Bodine et al., 2001). Disuse atrophy results in decrease in this signaling pathway by decrease the phosphorylation of Akt and its downstream target mTOR. This ultimately leads to a decreased formation of the translation initiation complex and therefore, muscle protein synthesis.

In summary, it is believed that increased protein degradation and decreased protein synthesis contribute to disuse muscle atrophy. Of note, the increase in protein degradation persists only for a few days, while the decrease in protein synthesis happens within hours and remains depressed for the duration of disuse.

## Endurance exercise training-induced adaptive response in skeletal muscle

Classic studies elegantly demonstrate that prolonged endurance exercise training results in beneficial adaptations to skeletal muscle (Saltin et al., 1977; Holloszy and Coyle, 1984). Note that resistance exercise may also have positive effects in skeletal muscle. Unless otherwise stated, exercise training will specifically reference prolonged moderate to high intensity endurance exercise training. One of the fundamental principles of endurance training is a beneficial changes that with mitochondria in skeletal muscle. Given than exercise intensity and duration are sufficient to elicit a response, mitochondrial alterations are two-fold. First, there is an increase in mitochondrial turnover that is due to both an increase in new mitochondrion being made and slight increase in old mitochondria being removed. The end result is that exercise creates a larger population of mitochondria within skeletal muscle. In addition, increase in mitochondrial content improves the control of energy metabolism, and results in the oxidation of more fatty acids and less glycogen for ATP production. Indeed, these alterations are important because they are in contrast of many of the changes that occur with disuse atrophy, and will be discussed in detail later in this review.

Many of the adaptations that occur with exercise training occur due to an adaptive response. In other words, acute intense exercise training imposes a brief physiological stress on skeletal muscle. Examples of these stresses include oxidative, thermal, mechanical, metabolic, and cytokine production. In response to these stressors, signaling pathways are activated in order to increase the protein content of stress response proteins. This review will focus on several proteins that may play a role in cytoprotection, which are increased in response to acute exercise training due to oxidative, metabolic, and thermal stress.

The notion that exercise training can be cytoprotective against disuse muscle atrophy originates from the idea that repeated bouts of the exercise over a prolonged period of time results in an accumulation of these cytoprotective proteins, and therefore allows the skeletal muscle to be more resistant to the stress. It is important to note that many of the signaling pathways and proteins that are increased following endurance exercise are also found to be increased in disease states. Disuse muscle atrophy or disease states are associated with chronic exposure to stressors, and therefore the alterations in signaling pathways and protein expression are required for maintenance of cell homeostasis. In contrast, exercise-induced cellular stress is acute, lasting at most a few hours. Rest periods following exercise allows the skeletal muscle to handle the stressor and ultimately adapt. In theory exercise training may protect muscle from disuse atrophy by providing a “reserve” of cytoprotective proteins and increases the cell's ability to resist stress.

## Role of mitochondria and oxidative stress in disuse muscle atrophy

Disuse muscle atrophy offers a different model of atrophy as opposed to many other models of atrophy because it is not associated with an increase in markers of inflammation. Therefore, the signals that promote atrophy are derived intracellularly. Evidence suggests that the major source of these intracellular signals arise in the mitochondria (Powers et al., 2012b).

### Mitochondrial morphology and function are altered during disuse

The first studies to demonstrate that muscle inactivity was associated with alterations in mitochondria were completed in the early 1960s. These studies demonstrated that muscles subjected to denervation had mitochondria that were irregularly shaped and swollen (Aloisi et al., 1960; Carafoli et al., 1964; Muscatello and Patriarca, 1968) and had impaired mitochondrial coupling. Since these initial investigations, it has become well accepted that disuse muscle atrophy results in altered mitochondrial morphology and mitochondrial dysfunction (Powers et al., 2012b). Indeed, disuse atrophy disrupts the functioning of the oxidative phosphorylation system (i.e., electron transport chain), which results in the generation of superoxide (O^−^_2_). The O^−^_2_ is dismutated to hydrogen peroxide and can then diffuse from the mitochondrion into the cytoplasm and negatively affect cell signaling pathways.

### The dual role of oxidative stress in disuse muscle atrophy and exercise-induced adaptations in skeletal muscle

Several studies have demonstrated that disuse atrophy promotes an increase in reactive oxygen species (ROS) production in skeletal muscle (Adhihetty et al., 2007b; Muller et al., 2007; Kavazis et al., 2009). This increase is accompanied by a decreased expression of mitochondrial proteins and decreased mitochondrial respiration (i.e., ATP production). Increased ROS production alters redox signaling in muscle fibers that can increase proteolysis and decrease protein synthesis (reviewed in Powers, 2014). To further compound these effects, disuse muscle atrophy is associated with an overall decrease in antioxidant capacity in skeletal muscle (Lawler et al., 2003; Falk et al., 2006; Kavazis et al., 2009; Min et al., 2011). This decrease in antioxidant capacity is likely due to decreased antioxidant scavenging ability and not a decrease in antioxidant enzyme content (Kondo et al., 1993; Lawler et al., 2003).

Paradoxically, it is well established that endurance exercise increases oxidative stress in skeletal muscle. The first report of exercise-induced ROS in skeletal muscle was first reported over 30 years ago by Davies et al. (1982). Since that seminal report, many studies have confirmed that muscle contraction markedly increases the amount of ROS production compared to resting skeletal muscle (Powers and Jackson, 2008). An increase in ROS production has been demonstrated to be a powerful signaling molecule in activating signaling pathways. Importantly, increased ROS production acts as a powerful signal for the activation of transcription factors that increase endogenous antioxidant proteins (Powers et al., 2011). Indeed, evidence now suggests that the transient oxidative stress that occurs with exercise may be required for skeletal muscle adaptation to occur. Studies in animals (Gomez-Cabrera et al., 2005, 2008a) and humans (Ristow et al., 2009) have demonstrated that preventing exercise-induced oxidative stress blunted an increase in markers of mitochondrial biogenesis and endogenous antioxidants with training. Therefore, it appears that exercise-induced oxidative stress is required for the adaptive response of antioxidants.

The key difference between exercise-induced oxidative stress and oxidative stress associated with disuse atrophy is the duration of the exposure. Oxidative stress following exercise is transient in nature, while disuse atrophy results in chronic exposure to elevated ROS. Therefore the next section will briefly discuss endogenous antioxidants that are important in maintaining homeostasis in skeletal muscle and will discuss which of these antioxidants may confer protection due to exercise preconditioning.

## The role of endogenous antioxidants in protection against disuse atrophy

Skeletal muscle maintains a tightly controlled network of antioxidant defense mechanisms to reduce the potential for oxidative damage during periods of increased oxidative stress. Indeed, prolonged muscle inactivity is associated with a decrease in antioxidant capacity as well as an increase in ROS production and emission from mitochondria (Lawler et al., 2003; Falk et al., 2006; Kavazis et al., 2009; Min et al., 2011). In this section, we will provide a brief overview of three primary antioxidant enzymes in muscle: superoxide dismutase, glutathione peroxidase, and catalase and three accessory antioxidant enzymes: thioredoxin, glutaredoxin, and peroxiredoxin.

### Superoxide dismutase

Three isoforms of superoxide dismutase (SOD) are present in skeletal muscle. All three isoforms convert superoxide radicals into hydrogen peroxide and oxygen using a transition metal. SOD1 primarily is located within the cytosol and the mitochondrial intermembrane space and requires copper-zinc (Cu,-Zn-SOD) as a cofactor. SOD2 uses manganese (Mn-SOD) and is located only in the mitochondrial matrix. SOD3 also uses copper-zinc as a cofactor and is distributed in the extracellular space.

The general consensus in the literature is that chronic exercise training results in an overall increase in total SOD activity (reviewed in Powers et al., 2011). However, which specific isoform(s) are primarily responsible for the increase in activity is less clear. To further complicate matters, some studies have shown different responses in mRNA, protein content, and enzyme activity following exercise. For example, Strobel et al. demonstrated that 14 weeks of exercise training in mice resulted in no change in SOD2 mRNA, an increase in SOD2 protein content, and an unexplained decrease in SOD2 activity in skeletal muscle (Strobel et al., 2011). The variability in responses can likely be explained by experimental differences, such as differences in exercise protocols, muscle groups studied (i.e., fiber type), timing of tissue sampling, and under-powered studies. Nonetheless, generalizations can be made for each isoform.

SOD1 and SOD2 are the primary isoforms that have been studied to date. In regard to SOD1, numerous studies have shown an increase in either mRNA expression, protein content, or enzymatic activity (Oh-Ishi et al., 1996, 1997; Gore et al., 1998; Gomez-Cabrera et al., 2008a,b; Ristow et al., 2009; Meier et al., 2013). However, a similar number of studies can be found that have reported no change in at least one of these markers (Oh-Ishi et al., 1996; Lambertucci et al., 2007; Brooks et al., 2008). Therefore, since evidence exists both for and against, it is difficult to determine if an increase in SOD1 following exercise can be protective. There is a similar body of literature for SOD2; however, the majority of studies demonstrate at least one marker of SOD2 (i.e., mRNA expression, protein content, or enzymatic activity) is increased with exercise training (Oh-Ishi et al., 1997; Gore et al., 1998; Lambertucci et al., 2007; Gomez-Cabrera et al., 2008a; Ristow et al., 2009). To date, little is known about the response of SOD3 to exercise training.

### Glutathione peroxidase and catalase

Hydrogen peroxide generated by the SOD family of antioxidants remains a reactive compound that can generate free radicals and is considered cytotoxic. Both glutathione peroxidase (GPX) and catalase are important antioxidants because they catalyze reactions that reduce hydrogen peroxide (reviewed in Powers and Jackson, 2008). In muscle cells, GPX expression is sensitive to oxidative stress (Zhou et al., 2001) and the majority of the literature suggests that GPX activity is increased following acute and chronic exercise (Laughlin et al., 1990; Criswell et al., 1993; Powers et al., 1994; Gore et al., 1998; Itoh et al., 2004; Lambertucci et al., 2007; Ristow et al., 2009; Smuder et al., 2011; Mangner et al., 2013). In contrast, changes in catalase expression following exercise is less clear, however the consensus is that catalase activity is not increased following training (Oh-Ishi et al., 1997; Smuder et al., 2011; Mangner et al., 2013).

### Thioredoxin, glutaredoxin, and peroxiredoxin

In addition to the previously discussed antioxidant enzymes, skeletal muscle also contains several accessory enzymes that contribute directly or indirectly as antioxidants. The most important accessory antioxidant enzymes include thioredoxin, glutaredoxin, and peroxiredoxin.

The thioredoxin enzyme system is composed of both thioredoxin (Txn) and thioredoxin reductase (TxnRd). There are two isoforms of thioredoxin that are delineated from each other based on the cellular compartment in which they are found. Txn1 located in the cytosol and Txn2 found in the mitochondrial matrix (Arnér and Holmgren, 2000). Txn is an important disulfide reductase in the cell and therefore is important in maintaining proteins in their reduced state (Holmgren, 1989). Furthermore, it has recently been suggested that Txn2, in conjunction with glutathione (GSH), is an important regulator of hydrogen peroxide emission from mitochondria (Aon et al., 2012). Importantly, Txn protein content is decreased with hindlimb unloading (Matsushima et al., 2006). Unfortunately, there is a little evidence on the response of Txn1 or 2 following exercise training. However, recent reports suggest that TxnRd2, a necessary component in the system needed to catalyze the reduction (e.g., recycling) of Txn2, is increased in skeletal muscle following training (Fisher-Wellman et al., 2013).

Similar to Txn, glutaredoxin (Grx) is a thiodisulfide oxidoreductase that protects and repairs protein thiols during periods of oxidant stress (Holmgren, 1989). Grx protects protein thiols by the transfer of electrons from NADPH; this catalytic response is coupled with GSH and glutathione reductase. Three Grx isoforms are found in skeletal muscle. Grx1 is located in the cytosol and Grx2 and Grx5 are found in the mitochondria (Holmgren, 1989).

Peroxiredoxin (Prdx) is a peroxidase capable of reducing hydrogen peroxide and other hydroperoxides and peroxynitrate using electrons provided by thiol compounds (e.g., Txn) (Yin et al., 2012). There are six isoforms of Prdx; PrdxI, Prdx2, and Prdx6 are located in the cytosol whereas Prdx3 is located in the mitochondria (Yin et al., 2012). Prdx5 is located in both the cytosol and mitochondria whereas Prdx4 is found in the extracellular space (Yin et al., 2012).

The paucity of data on the response of accessory antioxidant enzymes to exercise training in skeletal muscle makes it difficult to make concrete statements on their role in the prevention of oxidative stress in skeletal muscle; however a few general conclusions can be reached. The Prdx isoforms are the most commonly studied group of enzymes following exercise training. Unfortunately, a clear pattern of expression following exercise or disuse does not exist. In general, Prdx expression is decreased with unloading in skeletal muscle; however exercise training does not consistently increase expression of any of the isoforms.

The Txn system is of interesting note because it has recently been reported that Txn2, in conjunction with glutathione (GSH), is an important regulator of hydrogen peroxide emission from mitochondria (Stanley et al., 2011; Aon et al., 2012). Importantly, Txn protein content is decreased with hindlimb unloading (Matsushima et al., 2006). Unfortunately, there is a little evidence on the response of Txn1 or 2 following exercise training. However, recent reports suggest that TxnRd2, a necessary component in the system needed to catalyze the reduction (e.g., recycling) of Txn2, is increased in skeletal muscle following training (Fisher-Wellman et al., 2013). Therefore, it stands to reason that exercise may prevent disuse atrophy-induced oxidative stress by increasing the buffering capacity of H_2_0_2_ in mitochondria.

In summary, endurance exercise training improves muscle antioxidant capacity as evidenced by several studies demonstrating less oxidative stress in trained muscles following a bout of intense exercise. The current body of literature suggests that the improvement in antioxidant capacity is due in part to increased activity of SOD2 and GPX, with the possibility that SOD1 may play a contributory role. Additional work is needed to clarify the importance and physiological roles that accessory antioxidant systems in regards to exercise training adaptations and disuse muscle atrophy, however promising new experiments suggest that these antioxidant systems may provide a novel approach to preventing disuse atrophy.

### Can increased antioxidants protect against disuse muscle atrophy?

It has been difficult to determine cause and effect for the hypothesis that increased antioxidant capacity can prevent disuse muscle atrophy. One reason for this is the lack of ability to use genetic knockout mice to determine gain or loss of function. SOD1 genetic knockout mice (SOD1^−/−^) display increased markers of oxidative stress and marked muscle atrophy compared to wild type controls (Muller et al., 2006). Overexpression of SOD1 results in motor neuron disease and gross atrophy of skeletal muscle (Gurney et al., 1994; Rando et al., 1998; Zhang et al., 2013). It is believed that this sensitization of muscle to increased SOD1 activity is due to an imbalance between hydrogen peroxide generated by SOD1 without a concomitant increase in the enzymes that convert hydrogen peroxide to water and oxygen. Moreover, disruption of the SOD2 gene is lethal in mice due to neurodegeneration and cardiac dysfunction (Lebovitz et al., 1996). Therefore, it is difficult to conclude that increased expression of SOD1 or SOD2 can protect against oxidative stress during periods of disuse.

Without the ability to use transgenic animals, a common experimental design to determine the effect of antioxidant capacity on disuse muscle atrophy has been to provide exogenous antioxidant supplements. Examples of these supplements include vitamin C, vitamin E, trolox, N-acetylcysterine, and mitochondrial targeted antioxidants. Unfortunately, definitive proof that antioxidant supplementation protects against disuse atrophy does not exist because there is data for and against the idea that antioxidants can prevent disuse atrophy. For example, several studies using a variety of antioxidants in various disuse atrophy models suggest that antioxidant supplementation may provide protection against disuse atrophy (Kondo et al., 1991, 1992; Appell et al., 1997; Betters et al., 2004; Servais et al., 2007; Agten et al., 2011; Min et al., 2011; Talbert et al., 2013b), while several other papers provide evidence that antioxidant supplementation is not protective (Koesterer et al., 2002; Farid et al., 2005; Brocca et al., 2010; Desaphy et al., 2010). Indeed, these studies have spurred debates if oxidative stress is a cause of muscle atrophy or purely a consequence (Brocca et al., 2010; Powers et al., 2012a); compelling arguments for each side can be made. It is clear that more research is needed in this area, with a particular focus to address this issue in humans.

## The role of mitochondria and PGC1-α in disuse muscle atrophy

**Figure 2:**
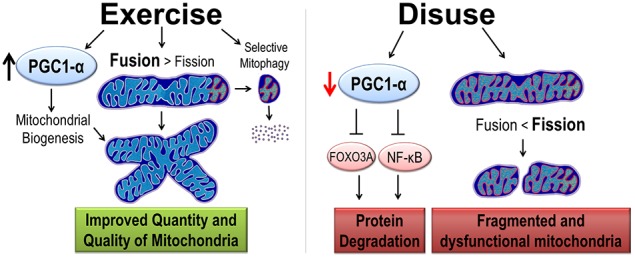
**Exercise and disuse effects on mitochondrial quality and quantity**. Exercise training promotes cytoprotective effects by the induction of PGC1-α protein, considered the master regulator of mitochondrial biogenesis. Exercise improves quality in two ways. First, mitochondrial dynamics are shifted such that fusion is greater than fission resulting in larger mitochondrial network. Second, increased selective mitophagy removes old and dysfunctional mitochondria (denoted with red and orange organelles). In contrast, disuse decreases PGC1-α, removing its inhibition of protein degradation pathways. Furthermore, mitochondrial dynamics are shifted toward fission causing a small, fragmented, and dysfunctional mitochondria.

As previously mentioned, exercise training and disuse have opposite effects on skeletal muscle mitochondria. Prolonged exercise training results in an increase in mitochondrial turnover. However, because mitochondrial biogenesis is increased at a greater rate than removal of mitochondria, exercise training results in an increase in an overall increase in mitochondrial content. A potent activator of mitochondrial biogenesis is peroxisome proliferative activated receptor-γ coactivator 1α (PGC1-α) (Figure 2). Thus, the first part of this section will focus on PGC1-α's role in mitochondrial turnover followed by a discussion on PGC1-α regulation of protein degradation.

### PGC1–α increases mitochondrial content is skeletal muscle

PGC1-α was first discovered in the lab of Bruce Spiegelman while studying adaptive thermogenesis in adipose tissue and skeletal muscle (Puigserver et al., 1998). This study speculated that increased expression of PGC1-α was an important modulator of mitochondrial biogenesis. Indeed, follow-up studies in skeletal muscle demonstrated that PGC1-α acts as co-activator by docking with transcription factors and aiding in the recruitment of regulatory protein complexes that increase gene transcription of proteins involved in mitochondrial biogenesis, rates of cellular respiration, and glucose uptake/utilization (Puigserver et al., 1998, 1999; Wu et al., 1999; St-Pierre et al., 2003).

In skeletal muscle, PGC1-α is frequently considered a “master regulator” of mitochondrial biogenesis because of its powerful role as a transcription co-factor. Indeed, PGC-1α interacts with and co-activates numerous transcription factors and nuclear receptors, including estrogen-related receptor-α (ERRα), nuclear respiratory factor-1 (NRF-1) and 2, myocyte enhancer factor 2 (MEF2), and mitochondrial transcription factor A (Tfam) (Spiegelman, 2007). All of these transcription factors directly regulate the expression of important nuclear-encoded mitochondrial genes necessary metabolic adaptations in skeletal muscle. Furthermore, overexpression of PGC1-α drives skeletal muscle fiber types to much more oxidative phenotype (Lin et al., 2002).

### Mitochondrial and metabolic alteration with disuse and exercise

Prolonged disuse muscle atrophy is associated with impaired oxidative metabolism and an increase in glycolysis. This is due in part to a decrease in mitochondrial biogenesis, mitochondrial content, and electron transport chain components (Lecker et al., 2004; Kang and Ji, 2013). The onset of mitochondrial dysfunction is rapid and results in an increase in ROS production and metabolic stress due to increased reliance on glycolysis, decreased fat oxidation, and substrate accumulation, which ultimately leads to inefficient ATP production (Stein and Wade, 2005). The opposite is true for exercise trained muscle, which demonstrates an increase in oxidative metabolism, increased utilization of fat, and increased mitochondrial biogenesis (Holloszy and Coyle, 1984). It is becoming increasingly apparent that PGC1-α is an important mediator of these exercise-induced effects and may be an important mediator of exercise induced-cytoprotection. Because PGC1- α is such a power inducer of fuel intake and β-oxidation, it follows that the metabolic changes that occur with exercise-induced PGC1- α expression can protect against metabolic deficits seen with disuse atrophy.

### PGC1-α activity regulates protein degradation pathways

Although it is well established that the decrease in protein synthesis contributes to disuse atrophy; to date, there has been no data suggesting that PGC1-α signaling is directly mediates the protein synthesis pathways (Brault et al., 2010). However, PGC1-α transcriptional activity has been shown to prevent muscle protein degradation. This was first demonstrated by Sandri et al., who showed that over-expression of PGC1-α in mice prevented denervation-induced muscle atrophy by preventing the expression of key genes in the ubiquitin proteasome pathway and autophagy (Sandri et al., 2006). Furthermore, this study demonstrated that elevated PGC1-α content prevented transcriptional activity of FoxO3a. Upon activation, FoxO3a moves from the sarcoplasm into the nucleus where it acts as a potent translational activator of proteins in the ubiquitin-proteasome (i.e., atrogin-1 and MAFbx), autophagy, and apoptosis pathways. Additionally, a recent study suggests that PGC1-α can inhibit the NF-κ B signaling pathway (Eisele et al., 2013), which is another important pathway in muscle protein degradation (reviewed in Jackman and Kandarian, 2004). Therefore, we conclude that PGC1-α is an important modulator of protein degradation pathways in disuse muscle atrophy and thus helps mediated the exercise preconditioning effect (Figure 2).

The rationale that PGC1-α mediates exercise-induced cytoprotection is two-fold. First, disuse muscle atrophy is associated with a decreased expression of PGC1-α content, as demonstrated in both animal and human models (Sandri et al., 2006; Sacheck et al., 2007; Kang and Ji, 2013). Therefore, it stands to reason, that preventing a decrease below basal levels of PGC1-α may prevent muscle atrophy. In this regard, PGC1-α expression is highly inducible with high intensity endurance exercise (Baar et al., 2002; Pilegaard et al., 2003; Smuder et al., 2012a) and importantly protein expression accumulates in skeletal muscle after training (Russell et al., 2003; Taylor et al., 2005). Thus, it follows that exercise training, prior to disuse muscle atrophy, can increase PGC1-α protein content such that the inactivity-induced decrease is mitigated or it only reaches the level of the basal untrained state. Indeed, expression of PGC1-α in fast-twitch muscles of mice, to levels commonly seen with exercise training, were able to attenuate disuse muscle atrophy (Sandri et al., 2006); thus, making PGC1-α a potentially important mediator of disuse atrophy protein degradation.

## Endurance exercise improves mitochondrial quality

As previously mentioned, exercise training is a potent stimulus for mitochondrial biogenesis; however, recent evidence suggests that endurance training may also improve mitochondrial quality through two mutually exclusive pathways: alterations in mitochondrial dynamics and selective autophagic degradation of mitochondria (i.e., mitophagy).

### Mitochondrial dynamics

Isolated mitochondria are often represented as spherical shaped individual organelles, however, *in vitro* they form a complex interconnected network. The shape of the mitochondrial network is said to be dynamic because segments of mitochondria frequently divide (i.e., fission) and join (i.e., fusion). Mitochondrial dynamics are controlled by a family of mitochondrial shaping proteins (Dimmer and Scorrano, 2006; Iqbal and Hood, 2014). Key proteins that promote mitochondrial fission include Dynamin-related protein-1 (Drp1) and Fission 1 (Fis1), whereas proteins regulating mitochondrial fusion include optic atrophy 1 (Opa1) and mitofusion 1 and 2 (Mfn1/2) (reviewed in Westermann, 2010; Chan, 2012). The role of fusion and fission in cells is complex, but it is believed that increased fusion of mitochondria is associated with improved function, while fission is a mechanism by which damaged portions of mitochondria are sectioned off the reticulum and targeted for degradation by autophagy (i.e., mitophagy).

### Mitophagy

As previously discussed, autophagy is the degradation pathway that allows cells to degrade organelles and macromolecules. There are two classifications of autophagy, selective or non-selective (i.e., macroautophagy). Selective autophagy is defined by the autophagasome directly targeting an organelle that has been selected by the cell to be degraded. Often, selective autophagy refers to degradation of mitochondria or endoplasmic reticulum. Non-selective autophagy indiscriminately degrades organells and macromolecules. The process of autophagy consists of cytosolic components to be degraded that are sequestered into double membrane vesicles called autophagosomes. These autophagosomes fuse with lysosomes containing hydrolytic enzymes that break down cellular components (He and Klionsky, 2009). Although autophagy plays a prominent role in protein degradation during disuse, it also appears to have a housekeeping role by turning over organelles and degrading protein aggregates. Mice with genetic ablation of rate limiting proteins in the autophagic machinery display marked muscle atrophy and weakness (Masiero et al., 2009; Chang et al., 2012).

Mitophagy denotes the degradation of mitochondria through autophagy and is regulated independently of macroautophagy (Kanki and Klionsky, 2008). Following damage, the mitochondrial phosphatase and tensin homolog-induced kinase-1 (PINK1) becomes stabilized on the outer mitochondrial membrane (Vincow et al., 2013). PINK1 then recruits the protein Parkin, an e3 ubiquitin ligase, which tags several outer mitochondrial membrane proteins with ubiquitin and results in the fragmentation and isolation of impaired mitochondria (Narendra et al., 2010). Currently, little is known about the proteins that interact between the autophagasome and PINK1 and Parkin on the mitochondria. Briefly, several candidate proteins have been identified. For example BCL-2, a key anti-apoptotic factor, binds to proteins related to phagasome development (Liang et al., 1999). BNIP3 (Bcl-2 and 19 kD interacting protein-37) and BNIP3-like protein (BNIP3L) have also been identified as proteins than can pair autophagasomes with uqiquintated mitochondrial protein (Zhang and Ney, 2009). Finally, autophagy related gene 32 (Atg32) is another protein recently to confers selectivity of the autophagosome to mitochodnria (Kanki et al., 2009). The field of mitophagy is an active area of research that is rapidly evolving and complex field of study, and therefore the specifics are beyond the scope of this review. Please see the following references for in depth review (Twig and Shirihai, 2011; Youle and Narendra, 2011; Feng et al., 2013).

### Mitochondrial dynamics and mitophagy in exercise and disuse

Mitochondrial dynamics and mitophagy are both altered with exercise training (Figure 2). In regard to mitochondrial dynamics, George Brooks' lab in the late 1980s was the first to demonstrate that the mitochondria reticulum became more expansive following chronic exercise training (Kirkwood et al., 1987). This suggested exercise training shifted the expression of fission and fusion machinery toward that of enhanced fusion. Improvements in biochemical and imaging techniques have provided evidence to support this theory. Exercise training is sufficient to increase expression of MFN1/2 and increases interactions between mitochondria (Ding et al., 2010; Picard et al., 2013; Iqbal and Hood, 2014). A primary pathway that drives the expression following exercise is PGC1 (Cartoni et al., 2005; Zechner et al., 2010) Concomitantly, fission protein expression is reduced with training (Perry et al., 2010; Iqbal and Hood, 2014). So what is the role of increased fusion of mitochondria? It is believed that the physiological relevance of increased fusion is mixing of mitochondrial content such as metabolic substrates, mitochondrial DNA, and proteins. Therefore, content mixing allows for a homogenous population of mitochondria, which in theory allows healthy mitochondria to positively affect damaged mitochondria.

Disuse atrophy is associated with a fragmented mitochondrial reticulum that is remodeled through autophagy (Romanello et al., 2010) (Figure 2). Note, mitochondria often appear enlarged in skeletal muscle atrophy (Powers et al., 2012b). This is not to be confused with a smaller, disorganized network. Instead, these mitochondria are enlarged due to swelling. Disuse atrophy results in an imbalance between fission and fusion events with an overall shift in the direction of fusion. Based on protein expression levels, disuse atrophy results in a down regulation of fusion machinery while fission events remain relatively constant (Iqbal and Hood, 2014; Cannavino et al., 2015). However, it is prudent to not exclude an increase in fission proteins with only a few data points. Indeed, expression of fission machinery is sufficient to cause muscle atrophy in mice (Romanello et al., 2010). Nonetheless, it is clear that disuse atrophy shifts the balance between fusion and fission results in small, fragmented mitochondria with a reduced capacity of ATP re-synthesis and increase in ROS emission (Twig et al., 2008).

It has recently been demonstrated that exercise training induces mitophagy, which is required for some of the exercise induced adaptations of skeletal muscle (Grumati et al., 2011; Lira et al., 2013). Although slightly counter intuitive, increased mitophagy following exercise is beneficial by improving overall mitochondrial quality through the selective removal of damaged or dysfunctional mitochondria (Safdar et al., 2011). Combined with evidence that exercise training or chronic stimulation of muscle results in a mitochondrial phenotype that resists oxidative stress and mitochondrial mediated apoptosis (Adhihetty et al., 2007a; O'leary and Hood, 2008), these data suggest that exercise training induced global changes in mitochondria may be cytoprotective by increasing quantity and improving quality of skeletal muscle mitochondria (Figure 2).

As previously discussed, disuse atrophy causes a decrease in mitochondrial number through targeted degradation. Mitochondria are segregated by fission and then selectively targeted for degradation via mitophagy. This process is to remove damaged mitochondria and decrease mitochondrial number to match metabolic demands. Therefore, mitophagy, *per se*, is not a direct contributor to disuse atrophy.

In summary, it appears that a basal level of mitophagy is necessary to maintain cellular homeostasis. The function of mitophagy is to remove damaged and/or old organelles so that these can be replaced by newer, healthier organelles. Exercise training is the excellent example of mitophagy improving overall mitochondrial quality by increasing following exercise training and remodeling of the mitochondrial phenotype.

## The role of heat shock protein 70 in prevention of muscle atrophy

The heat shock family of proteins is known as molecular chaperone proteins that have been demonstrated to increase following exercise. In general, their role is three-fold: (1) facilitate folding of de novo synthesized proteins; (2) assist in the re-folding of misfolded proteins and denatured proteins, and (3) aid in the transport of those proteins to their correct cellular compartments (Feder and Hofmann, 1999). Heat shock proteins (HSPs) can be classified into a number of families that are traditionally named for their molecular mass. These include small HSPs (between 8 and 27 kDa), HSP60 (i.e., 60 kDa), HSP 70, and HSP90. Of these, HSP70 is the most studied and will be the focus of the following section.

### HSP70 is induced by cell stress and exercise

HSP70, also commonly referred to as HSP72, is a highly conserved protein whose expression is increased following stress, hence why HSP70 is termed a stress-inducible member of the HSP family of proteins. Its expression is responsive to thermal stress, oxidative stress, mechanical stress, metabolic stress, and cytokines. Importantly, exercise training promotes these stressors and it is clear that exercise is a powerful inducer of HSP70 protein content.

The first evidence that exercise increased HSP70 protein content was generated in 1990 when Locke et al. postulated that exercising skeletal muscle from mice increased protein synthesis of stress protein similar to that of a protein produced during heat stress (Locke et al., 1990). Shortly thereafter, it was confirmed that exercise training increased HSP70 mRNA in both rodent (Salo et al., 1991) and human skeletal muscle (Puntschart et al., 1996). Since these original studies, it is now well accepted that a single bout of exercise is sufficient to increase HSP70 mRNA and protein expression in animals and humans in an intensity dependent manner (Milne and Noble, 2002; Morton et al., 2009). Importantly, prolonged exercise training increases baseline levels of HSP70 (Liu et al., 1999; Ecochard et al., 2000; González et al., 2000; Atalay et al., 2004; Kayani et al., 2008; Smuder et al., 2012b). It is important to note that mechanical unloading has the opposite effect of exercise on HSP70 content in muscle. Disuse atrophy is associated with a rapid decrease in HSP70 protein content (Lawler et al., 2006; Senf et al., 2008; Moriggi et al., 2010; Lomonosova et al., 2012).

### Increased HSP70 content protects against disuse muscle atrophy

Early studies demonstrated that exposing mice to a non-lethal heat stress provides the animal with protection against a subsequent typically lethal heat exposure. These protective findings were attributed to a family of proteins that were induced by heat stress and these findings were soon translated to skeletal muscle (Garramone et al., 1994). In the study by Garramone et al., prior exposure to heat stress (i.e., greater than 41°C) conferred protection against ischemic injury in rat skeletal muscle (Garramone et al., 1994).

In regards to disuse muscle atrophy, there are several lines of evidence that induction of HSP70 content can protect against the negative biochemical and structural changes associated with muscle atrophy. First, prior exposure to heat stress demonstrates protection against atrophy in hind limb unloading, immobilization, and mechanical ventilation (Naito et al., 2000; Selsby and Dodd, 2005; Ichinoseki-Sekine et al., 2014). However, because heat stress may cause other adaptive responses independent of HSP70, these studies do not prove cause and effect. Direct evidence in support of this hypothesis comes from muscle-specific overexpression of HSP70. For example, Senf et al., demonstrated that HSP70 overexpression attenuated 4 days of immobilization-induced muscle atrophy (Senf et al., 2008). However, conversely, another study using whole body overexpression was unable to prevent 7 days of unloading-induced atrophy (Miyabara et al., 2012). Several possibilities exist for this discrepancy including species differences (rat/mice), duration of immobilization (4 day/7 days), and method of overexpression (single muscle electroporation/whole body overexpression). It important to note that the global (i.e., whole body) HSP70 overexpressing mice had significantly smaller muscles compared to their wild type littermates, suggesting altered physiology in these animals (Miyabara et al., 2012). Nonetheless, it appears that HSP70 is an important stress response protein that has the potential to protect against disuse muscle atrophy.

### Mechanisms by which HSP70 protects against muscle atrophy

**Figure 3:**
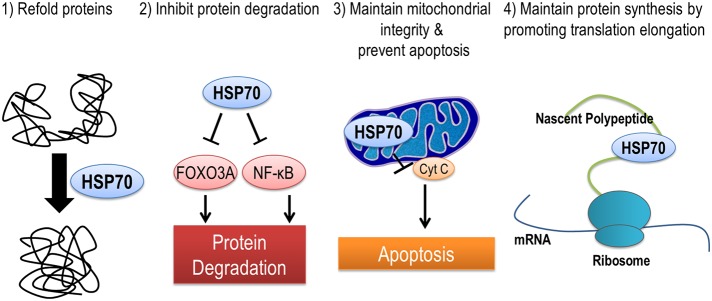
**HSP70 is an inducible chaperone protein that can be provide protection against muscle atrophy in several ways**. (1) Refolding of misfolded or unfolded proteins, (2) inhibiting select pathways of protein degradation, (3) maintaining mitochondrial integrity and reducing apoptosis by preventing the release of cytochrome C from mitochondria, and (4) improving translation elongation and promoting protein synthesis.

HSP70 is localized in many different subcellular locations including mitochondria, nucleus, ribosomes, and in the cytoplasm. Therefore, it is no surprise that HSP70 may protect against muscle atrophy through a multitude of different mechanisms (Figure 3). First, HSP70 is most well-known for its ability to serve as a chaperone and prevent dysfunction and/or targeted proteolysis of oxidized proteins by binding to and assisting in their re-folding. An important example of this mechanism is demonstrated by the ability of HSP70 to protect against damaging increases in intracellular calcium during muscle atrophy by binding to oxidized sarcoplasmic/endoplasmic reticulum Ca^2+^-ATPase (SERCA) to preserve its function (Gehrig et al., 2012). Second, the chaperone function of HSP70 can maintain mitochondrial integrity by protecting mitochondria against apoptotic stimuli and contribute to the repair of damaged proteins (Kang et al., 1990; Mosser et al., 2000). Third, HSP72 can prevent disuse atrophy induced-proteolysis by inhibiting activation of FoxO3a and NF-κ B signaling pathways (Senf et al., 2008, 2010). And finally, expression of HSP72 may prevent an atrophy induced decrease in muscle protein synthesis by maintaining the rate of translation elongation of nascent polypeptides (Nelson et al., 1992; Ku et al., 1995).

## Conclusions

The idea that lifelong endurance training can be protective against disuse muscle atrophy has yet to be definitively proven in scientific literature, however the theory remains valid. An acute bout of exercise training results in a small stimulus that brings about an adaptive response. Prolonged exercise training results in an accumulation of these adaptive responses. Therefore, it follows that when skeletal muscle has adapted to small repeated stresses, it is much better suited to handle the stress brought on by disuse muscle atrophy. These adaptations include, but are not limited to, limiting oxidative stress during disuse atrophy by increased antioxidant systems to buffer increased ROS production or by having newer, healthier mitochondria that produce less ROS when inactive. There is also the possibility of increased proteins such as PGC1-α and HSP70 that can prevent activation of key protein degradation pathways, protect against mitochondrial dysfunction, maintain muscle protein synthesis, and assist in the refolding of damaged proteins. Furthermore, there are likely numerous other proteins that could be cytoprotective and therefore more work in this area needs to be done.

### Conflict of interest statement

The author declares that the research was conducted in the absence of any commercial or financial relationships that could be construed as a potential conflict of interest.
